# A Taxonomy of Food Supply Chain Problems from a Computational Intelligence Perspective

**DOI:** 10.3390/s21206910

**Published:** 2021-10-18

**Authors:** Juan S. Angarita-Zapata, Ainhoa Alonso-Vicario, Antonio D. Masegosa, Jon Legarda

**Affiliations:** 1 Deusto Institute of Technology (DeustoTech), Faculty of Engineering, University of Deusto, 48007 Bilbao, Spain; ainhoa.alonso@deusto.es (A.A.-V.); ad.masegosa@deusto.es (A.D.M.); jlegarda@deusto.es (J.L.); 2 Ikerbasque, Basque Foundation for Science, 48009 Bilbao, Spain

**Keywords:** food supply chain, computational intelligence, fish farming, agriculture, livestock, machine learning, neural networks, deep learning, meta-heuristics, fuzzy systems, probabilistic methods

## Abstract

In the last few years, the Internet of Things, and other enabling technologies, have been progressively used for digitizing Food Supply Chains (FSC). These and other digitalization-enabling technologies are generating a massive amount of data with enormous potential to manage supply chains more efficiently and sustainably. Nevertheless, the intricate patterns and complexity embedded in large volumes of data present a challenge for systematic human expert analysis. In such a data-driven context, Computational Intelligence (CI) has achieved significant momentum to analyze, mine, and extract the underlying data information, or solve complex optimization problems, striking a balance between productive efficiency and sustainability of food supply systems. Although some recent studies have sorted the CI literature in this field, they are mainly oriented towards a single family of CI methods (a group of methods that share common characteristics) and review their application in specific FSC stages. As such, there is a gap in identifying and classifying FSC problems from a broader perspective, encompassing the various families of CI methods that can be applied in different stages (from production to retailing) and identifying the problems that arise in these stages from a CI perspective. This paper presents a new and comprehensive taxonomy of FSC problems (associated with agriculture, fish farming, and livestock) from a CI approach; that is, it defines FSC problems (from production to retail) and categorizes them based on how they can be modeled from a CI point of view. Furthermore, we review the CI approaches that are more commonly used in each stage of the FSC and in their corresponding categories of problems. We also introduce a set of guidelines to help FSC researchers and practitioners to decide on suitable families of methods when addressing any particular problems they might encounter. Finally, based on the proposed taxonomy, we identify and discuss challenges and research opportunities that the community should explore to enhance the contributions that CI can bring to the digitization of the FSC.

## 1. Introduction

Currently, one worldwide challenge is how to sustainably guarantee global food needs in the face of a growing population that is projected to be 9–10 billion by 2050 [1]. In this sense, the enhancement of production and management of the current Food Supply Chains (FSCs) is a crucial factor that contributes to accomplishing such an aim. Nowadays, new Information and Communication Technologies (ICTs) (e.g., the Internet of Things) play an active role in the digitization of FSCs [2]. As a result, large volumes of data are being generated in all FSC stages, ranging from production to retail. The analysis of such data would enable FSC actors to extract relevant information or to optimize specific processes, allowing improvement of the FSC administration, productivity, and sustainability.

Nevertheless, the high volumes of available data and their complex patterns raise significant challenges when analyzing and extracting values. In this context, Computational Intelligence (CI) seems to be a successful paradigm to build intelligent systems that are able to leverage this high availability of data. CI is the ability of a digital system or algorithm to perform tasks commonly associated with intelligent beings [3]. Within such tasks, we can find speech recognition, visual perception, decision-making, prediction, and translation, among others [4]. Over the last few years, the number of academic publications concerning CI applied to FSC has rapidly increased [5,6,7]. Within the most representative CI methods applied to FSCs, we find Neural Networks, Fuzzy Logic, Swarm Intelligence, or Probabilistic Reasoning.

The scientific literature reports different studies that aim to review and order the application of CI methods in different FSC stages. The variety of CI methods has led to the emergence of research papers (published between 2012 and 2020), which select a particular family of CI techniques and review their application in specific FSC stages [2,6,7,8,9,10,11,12]. However, these papers focus on only one or two families of CI methods at most, and in the majority of cases, do not cover all FSC stages. Therefore, there is a lack of comprehensive studies that review the application of the most important families of CI methods in all FSC stages (from production to retail). Additionally, few efforts have been made to classify FSC problems from a CI perspective. Hence, there is no categorization of the typologies of FSC problems to help determine how they can be modeled from a CI view (e.g., optimization, uncertain knowledge handling, reasoning) and what CI methods can be most suitably used to approach them. Thus, despite the progress made in organizing and systematizing the existing literature at the point where CI and FSCs meet, to the best of our knowledge, no taxonomy has been proposed in this regard.

With the above-mentioned ideas in mind, we propose a novel taxonomy of FSC problems from a CI perspective. Specifically, we focus on the supply chain of agriculture, fish farming, and livestock. The latter is justified based on the fact that these supply chains provide most of the food consumed by the population of the world [13] and, therefore, they are the most studied and researched FSCs in the scientific and academic literature. The main contributions of this article are:

- A new taxonomy that provides a comprehensive view of different FSC problems located in the chain stages typically studied in the scientific literature (production, processing, distribution, and retail). This taxonomy represents a new and broader proposal in order to identify and define FSC problems that have been approached using CI in the four aforementioned stages. Besides, although some research articles have described diverse FSC problems, their definitions are not unified and vary from one paper to another. Thus, this taxonomy also represents an effort to unify and consolidate definitions of the FSC problems available in the literature, which represents a valuable source of information for FSC researchers and practitioners working in this domain.
- To classify the FSC problems from a CI perspective. This classification allows FSC problems to be mapped into common categories of problems in the CI domain. Thus, we provide a framework that helps display the similarities and differences among FSC problems depending on how they can be modeled under a CI perspective. To the best of our knowledge, in this regard, no classification has been previously proposed.
- To establish a set of guidelines for the use of CI in the FSC field. These guidelines aim to help FSC researchers and practitioners to identify which FSC problems can be addressed using CI, and the most appropriate families of techniques to solve them. Thus, these guidelines represent a first attempt to define a general framework to support the model selection problem at the point where the fields of FSC and CI converge.
- To identify and discuss challenges and research opportunities in the FSC domain, which are directed towards more robust, explainable, interoperable, and accurate CI solutions that support FSC management and operation.

The rest of this paper is structured as follows. Section 2 provides background information on FSC and CI to facilitate the understanding of the article. Furthermore, it summarizes other efforts directed at reviewing and categorizing the scientific literature at the point where FSC and CI meet in order to highlight how this paper complements and enhances previous studies. Section 3 presents the proposed taxonomy, its structure, and the classification of FSC from a CI perspective. Afterwards, Section 4 gives FSC researchers and practitioners a set of guidelines for the use of CI within the FSC domain. Lastly, Section 5 summarizes the main conclusions and sets a research agenda for CI in the FSC field.

## 2. Background and Motivation

This section provides some contextual and relevant background information to facilitate the understanding of the paper, and to assess similar studies in this area. We start by introducing the basic FSC stages examined in this study (Section 2.1). Then, Section 2.2 presents the main families of CI approaches typically considered in FSC research. Finally, Section 2.3 reviews similar works at the point where FSC and CI converge, which have identified and classified CI-based problems and methods. Section 2 ends with a discussion of the main contributions of this research article.

### 2.1. Food Supply Chain

FSC refers to the system that encompasses all activities, organizations, actors, technologies, information, resources, and services involved in producing agri-food products for consumer markets (e.g., fresh food, meat, and processed food products) [14]. The upstream and downstream sectors form the supply of agricultural inputs (such as seeds, fertilizers, feed, medicine, or equipment) to production, post-harvest handling, processing, transportation, marketing, distribution, and retailing [15]. They also include support services such as extension services, research and development, and market information.

Before the final consumption of food products, FSC can include diverse stages wherein production, processing, distribution, and retail are the stages most commonly studied in the scientific literature [16]. First, the production stage forms the initial set of processes to obtain raw products derived from agriculture, fish farming, or livestock. Agriculture refers to farming applications, including the cultivation of soils for the harvest of crops [17]. Fish farming involves raising fish commercially in tanks or fish ponds, usually for consumer markets [18]. Meanwhile, livestock is devoted to practices related to animal husbandry for meat, milk, eggs, or wool [19]. Continuing further down the production chain, we find the processing stage, wherein the produced raw materials are exposed to diverse transformation processes (e.g., meat curing, washing and disinfecting vegetables, fermentation), which allow consumable food products to be obtained.

Once food products are ready to be delivered to the end-users, the following FSC stages are distribution and retail. Their main purpose is to connect the production and processing stages with the food users to complete the supply chain loop [16]. Consequently, in the distribution stage, the processed food is sent to distribution centers and warehouses. From such locations, different distribution channels start to deliver the products to retail stores for sale. Thus, retail is the end stage of the FSC and represents the link to consumers.

FSCs consist of a wide range of enterprises, ranging from smallholders, farmers’ organizations, co-operatives, and start-up companies to multinational enterprises through parent companies or their local affiliates [14,15]. In this context, FSC companies relate to each other through a variety of arrangements. Downstream companies in the supply chain may engage in various types of relationships with producers to secure access to agricultural products. They can impose standards and specifications on producers with little involvement beyond a buying contract. However, they can also become more actively involved, particularly through contract farming, to coordinate production and ensure quality and safety [14]. In the new circular chain schemes, this type of relationship becomes more complex, involving more than one enterprise from the chain in the decision-making process [20].

Currently, an appropriate and effective strategy to address this challenge is the digitization of FSCs, both of their internal processes and of their relationships with other actors in the chain [2]. Digitization has led to the generation of big volumes of data throughout the entire supply chain. The exploitation of such data would allow FSC actors to extract knowledge that could improve their internal processes in terms of productivity and sustainability, as well as that of the FSC as a whole. However, the data obtained through digitization usually contain complex and intricate patterns that stand out as diverse challenges for processing and analysis to extract value from it. Thus, CI arises as a solution that could leverage and mine the underlying patterns of such data in order to obtain the maximum value of the information, according to the analyses made.

### 2.2. Computational Intelligence Approaches

CI is centered on the ability of a computer or algorithm to learn specific tasks (e.g., pattern recognition, forecasting) from data that is typically related to experimental observations without human intervention [3]. In the context of FSC, diverse IoT devices and data management systems sense and gather such data, which they then deploy in each supply chain stage. After obtaining data, different CI approaches are used to process, analyze, and extract information. In this section, we introduce the relevant background information in relation to families of CI-based methods that are typically used in FSC applications. Following the guidelines and classification proposed in [3], we have grouped the CI methods into five families that are presented in more detail below. They are CI-based Statistical Learning Methods, Artificial Neural Networks and Deep Learning, CI-based Optimization Methods, Fuzzy Systems, and Probabilistic Reasoning. These groups of CI-based methods are presented below.

#### 2.2.1. CI-Based Statistical Learning Methods

Statistical Learning Methods, also known as Machine Learning (ML) methods are algorithms that are able to learn a specific task without being explicitly programmed. More formally, according to Mitchell [21], these types of methods learn from experiences *E*, related to a task *T*, and their performance is evaluated by a metric *P*. The performance in *T* improves according to *P*, with experience *E*. Classically, these methods can be classified according to the three basic learning approaches presented below: unsupervised learning, supervised learning, and reinforcement learning.

Unsupervised learning looks for patterns in data with no pre-existing labels. Its central approach is usually focused on organizing *X* data points into specific groups [22]. Data points that are in the same group should have similar properties, while data points in different groups should have highly different features. It is important to note that these potential groups are not previously defined and it is the purpose of unsupervised learning algorithms to discover them. Some examples of unsupervised learning methods are hierarchical clustering, k-means, anomaly detection techniques, among others.

Supervised learning is the other fundamental area of Statistical Learning Methods [23]. It consists of algorithms that learn a function (f:X↦Y) by training with a finite number of input-output pairs, *X* being the input domain and *X* the output co-domain. This learning stage can be seen as *E* in Mitchell’s definition [21], and the specific task *T* usually involves predicting an output given a new and unseen input [24]. Common families of methods that stand out in supervised learning are decision tree-based (e.g., Decision Tree, Extra Trees), instance-based (e.g., K-Nearest Neighbors), kernel-based (e.g., Support Vector Machine), or ensemble-based methods (Random Forest, AdaBoost).

Supervised learning problems can usually be divided into classification and regression [25,26]. In both cases, the basis is an input data-set, *X*, and their difference is the type of target variable, *Y*, to be predicted. In the classification case, *Y* is divided into discrete categories, while in regression, the aim is to predict continuous values. Standard classification problems can be either binary or multi-class problems [27]. In the former case, an instance can only be associated with one of two values: the positive or negative equivalent to 0 or 1; whereas, in multi-class problems, there are more than two classes under consideration. A multi-class problem means that a given instance belongs to one of the multiple possible categories. Diversely, a supervised regression problem [28] consists of finding a function that can predict, for a given example, a real value in R.

The third learning approach is reinforcement learning [29]. In this case, the focus is on developing a learning agent able to observe the environment and obtain some input from it. Then, the agent makes an action and it changes to a new environment, receiving an evaluation value (award or penalty) related to the action made. Unlike the unsupervised learning approach, the agent receives guidance from an external evaluation. Moreover, different to supervised learning, in reinforcement learning, the agent is provided with an evaluation value regarding the action made and not with a clear specification about the correspondence between input and output data. Relevant approaches within reinforcement learning are value-based [30], policy-based [31], and model-based [32].

Lastly, it is important to note that Artificial Neural Networks are not considered within this family of methods. We will treat them as a separate class of methods because of their important role in CI.

#### 2.2.2. Artificial Neural Networks and Deep Learning

Artificial Neural Networks (ANNs) are computing systems with an inner structure that is based on a set of connected units, named neurons, as they are inspired by a biological brain. Just as is the case for animals, wherein two neurons are connected by means of a link (synapses), the neurons of an ANN are connected through edges that transmit signals from one artificial neuron to another. The signals transferred between neurons are represented by real numbers, and the output of each neural unit is computed by some non-linear function of the sum of its inputs.

**Figure 1:**
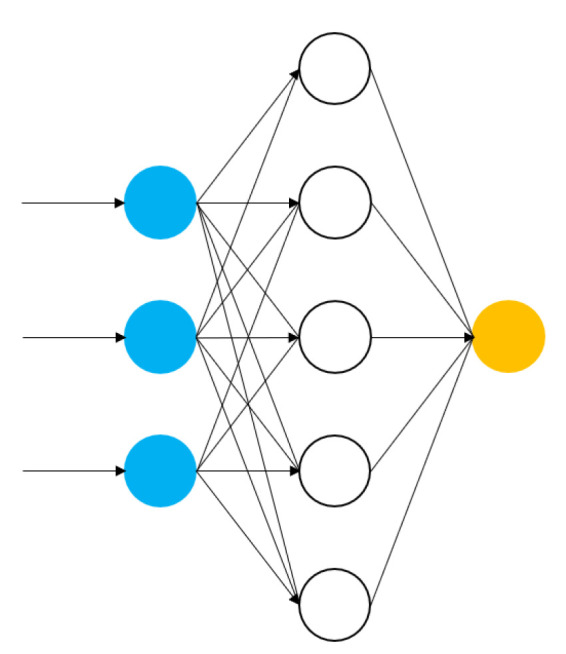
Basic architecture of an ANN. It is composed of edges and neural units arranged using an input layer (blue neural units), a hidden layer (white neural units), and an output layer (yellow neural units).

Every edge that connects two neurons (usually) has a weight that is adjusted as the learning process of the ANN is underway. The role of such edges is to increase or decrease the strength of the signal at the connection of two neurons. Additionally, neural units are aggregated into layers, and they may carry out diverse transformations on the inputs that they receive. Thus, the purpose of ANNs is to process input data from the first layer (input layer) to the last layer (output layer), while approximating linear or non-linear functions that are generally unknown. The collection of neuron units arranged in layers, edges, and weights forms a network topology that is usually called an architecture (Figure 1). ANNs have evolved into a broad family of architectures that depend on the specific application domain. The feed-forward neural network is a type of classical architecture. In this network, the input data moves from the input layer directly through any hidden layers (intermediate layers located in-between the input and output layers) to the output layer.

Because of their ability to reproduce and model nonlinear processes, ANNs have been applied in diverse supervised learning, unsupervised learning, and reinforcement learning problems. Application areas may include time series forecasting, pattern recognition, signal classification, among others. However, as more complex problems (e.g., image processing, speech recognition) have arisen in the last few years, further development in the ANN area is required to deal with resulting challenges. The common denominator of these fields is the high complexity and enormous volumes of data generated and managed in them. As a result, a subset of ANNs, named Deep Learning (DL) [33] has emerged to cope with this complexity.

A deep neural network is a classical ANN composed of multiple layers between the input and output layers. Theoretically, DL architectures allow any non-linear function to be approximated [34]. Therefore, this approach has become dominant in multiple application fields like computer vision [35] and natural language processing [36]. Between the most common deep neural networks, we can find recurrent neural networks, convolutional neural networks, and long short-term memory neural networks [37]. The main strength of this approach lies in its ability to learn automatically from raw data and to learn more complex representations of data than other Statistical Learning methods [38]. The latter could be valuable for research areas characterized by having complex data, which can barely be analyzed by human reasoning and classical data preprocessing approaches. The other relevant characteristics of Deep neural architectures come from their architectural flexibility that enables data fusion, as they allow different data formats to merge, combining data from multiple sources and therefore extracting more valuable knowledge. In other words, DL facilitates the use of multi-dimensional data, which is quite difficult to achieve with classical ANNs and ML methods.

#### 2.2.3. CI-Based Optimization Methods

The growing computational capabilities and the fact that some problems of great practical value (e.g., scheduling, routing, facility location) can not be solved optimally (because they are NP-Hard problems) has led to increased use of approximating algorithms. Meta-heuristics are a suitable approach in situations where exact algorithms can not give an answer using a reasonable amount of time or memory [39]. These methods arose with the idea of extracting the best parts of different successful heuristics to create generic methods that could be applied to a more significant number of problems and contexts. Due to the wide variety of meta-heuristics, different classification categories have been proposed [40]. We group these techniques according to the next categories: Evolutionary Computation (EC) [41], Swarm Intelligence (SI) [42], and other meta-heuristics (local search-based meta-heuristics [43]). These groups are presented with more detail as follows.

EC is a group of meta-heuristic optimization algorithms inspired by biological evolution. Within this family of methods, they operate from an initial set of candidate solutions (initial generation), which are updated in an iterative way. Then, each new generation is generated by randomly removing candidate solutions according to predefined criteria and by inserting random changes. After a set of iterations, the population of solutions will gradually evolve to increase its competitiveness, framed by a fitness function that is determined by each algorithm. Following the described procedure, EC algorithms can produce highly optimized solutions for complex real-world optimization problems like the traveling salesman problem [44]. Some well-known examples of EC meta-heuristics are Genetic algorithms [45] and Differential evolution [46], among others.

The second biggest category of meta-heuristics is the category of Swarm Intelligence (SI). This approach consists of a population of agents interacting with each other and their environment. The agents follow a set of basic rules, and although there is no centralized control structure guiding the agents on how they should behave, the interaction between them leads to the emergence of intelligent global behavior. SI is inspired by biological systems such as ant and bee colonies and is commonly used to solve combinatorial and continuous optimization problems (e.g., shortest path problems for delivery or optimization of unknown parameters in time series). Two of the most well-known SI algorithms are Ant Colony [47], and Particle Swarm [48] methods.

The third category groups together the rest of the meta-heuristics, which are outside of the domains of EC and SI but are still relevant for solving optimization problems. For example, this is the case for local search-based meta-heuristics that are focused on finding a solution that maximizes a criterion among a set of candidate solutions. These meta-heuristics move from one solution to another in the search space of candidate solutions by applying local changes until an optimal solution is found or a time budget is reached. Within this category of meta-heuristics, representative methods include Tabu Search [49], and Greedy Randomized Adaptive Search Procedure (also known as GRASP) [50].

#### 2.2.4. Fuzzy Systems

Classical logic is based on the crisp set concept, where a group of objects is considered to be a collection. In this sense, a crisp subset can be defined from a broader set where its elements belong to the subset according to some particular condition. Thus, we can define the concept of membership wherein a value of one is assigned to the elements of the subset and a value of zero to the elements that do not belong to that subset. Unlike crisp sets, a fuzzy set allows partial belonging to a set through a degree of membership, denoted by a function μ, that maps all the elements in the set to a value in the real interval between zero and one [51]. Then, as in the crisp case, a value of zero means that the element under consideration does not belong to the set, and a value of one represents that the element belongs entirely to the set. However, unlike in the crisp case, a value greater than zero and lower than one represents a partial membership to the subset. Consequently, the set’s membership function is the relationship between the elements of the set and their degree of belonging.

**Figure 2:**
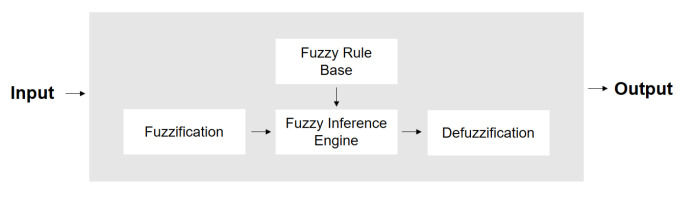
Structure and components of a Fuzzy System.

Having introduced the basic notions of fuzzy sets, the next key concept is fuzzy inference systems. A fuzzy system is a repository of fuzzy expert knowledge that can reason the data in vague terms instead of precise Boolean logic. This expert knowledge is a collection of fuzzy membership functions and a set of fuzzy rules that is formed as follows: IF (conditions are fulfilled) THEN (consequences are inferred). The basic configuration of a fuzzy system is shown in Figure 2, and it can be divided into four main parts: a fuzzifier, a knowledge base, an inference engine, and a defuzzifier [52].

The Fuzzifier maps a real crisp input to a fuzzy function and, therefore, determines the degree of membership of the input to a vague concept (categories using the fuzzy sets). The values of the input variables are mapped to the range of values of the corresponding universe of discourse. The range and resolution of input-fuzzy sets and their effect on the fuzzification process are considered to be factors that affect the overall performance of the system.

The knowledge base comprises the knowledge of the application domain. It can be split into a database of definitions used to express linguistic control rules in the controller and a rule base that describes the knowledge held by the experts in the domain. Intuitively, the knowledge base is the core element of a fuzzy controller as it will contain all the information necessary to accomplish its execution tasks.

The Inference Engine provides the decision-making logic of the controller. It deduces the fuzzy control actions by employing fuzzy implications and fuzzy rules of inference. In many aspects, it can be viewed as an emulation of human decision-making. Finally, the Defuzzification process converts fuzzy control values into crisp quantities; that is, it links a single point to a fuzzy set, given that the point belongs to the support of the fuzzy set. There are many de-fuzzification techniques, the most famous being the center-of-area or center-of-gravity.

#### 2.2.5. Probabilistic Reasoning

Probabilistic Reasoning states that users can infer plausible models to explain input data. Thus, a model can predict an output based on new, unknown input data, which allows decisions to be made regarding future actions. In this context of predictions, uncertainty plays a relevant role for three reasons [53]. First, uncertainty can be introduced from noisy input data into the training process of a model. Secondly, input data can be consistent with different models, and therefore which model is more appropriate for the data at hand is uncertain. And third, a model can have diverse parameters (e.g., the coefficients of linear regression) and/or different inner structures (e.g., the architecture of ANNs); hence, there is uncertainty regarding the specifications for a concrete model [54].

From a general perspective, the basic foundations of Probabilistic Reasoning are condensed in the Bayesian learning paradigm [54]. Primarily, probability distributions are considered to represent all uncertainties that can interfere in a model (e.g., noise in the input data, the model’s parameters). Then, the basic rules of probability theory are considered to infer unobserved quantities given the observed data. Thus, the process of learning from data occurs through the transformation of the prior probability distributions (defined before having the input data) into posterior distributions (after observing the data).

The assumptions mentioned above are supported by two of the basic rules of probability theory. They are the sum rule and the product rule, which can be expressed as $P\left(x\right)=\sum_{y\in Y}^{}P\left(x,y\right)$ and P(x,y)=P(x)P(y∣x), respectively. Here *x* and *y* correspond to observed or uncertain quantities, taking values in sets *X* and *Y*. P(x) is the probability of *x* regarding the frequency of observing a particular value. P(x,y) is the joint probability of observing *x* and *y*, and *P*(*y*|*x*) is the probability of *y* conditioned on observing a concrete *x* value. Keeping these two probability theory rules in mind, *x* and *y* can be integrated into the Bayes’ theorem to describe the probability of an event based on the prior knowledge of conditions that might be related to the event. In the context of Statistical Learning, this theorem is stated as $P\left(\theta |D,m\right)=\frac{P(D|\theta ,m)P(\theta |m)}{P(D|m)}$. Here, P(D∣θ,m) is the likelihood of parameters θ in model *m*, P(θ∣m) is the prior probability of θ, and P(θ∣D,m) is the posterior probability of θ given data *x*.

Thus, learning is the transformation of prior knowledge or assumptions regarding the parameters P(θ∣m), through data *D*, into the posterior knowledge about the parameters P(θ∣D,m). Such a posterior distribution then becomes prior knowledge for future data predictions. Within this framework, the most typical methods used over the last few years were Bayesian networks [55]. Other representative techniques are Markov networks and Random Fields [56].

#### 2.2.6. Summary of CI-Based Approaches

Having presented the families of CI methods usually considered in FSC, this section introduces a summary of the methods presented above and their strengths and weaknesses (Table 1). First of all, we would like to point out that this list of advantages and disadvantages does not refer to a comparison between the different categories of methods considered in this paper, as they are often used to solve different types of problems and therefore the comparison is not straightforward. Instead, these strengths and weaknesses refer to a comparison between CI-based approaches versus non CI-based approaches that are used to solve similar categories of problems.

### 2.3. Motivation

The objectives of this section are two-fold. First, it reviews the related work at the point where FSC and CI meet, in order to identify previous contributions regarding the classification of FSC problems, and the CI methods used to solve them. Having already introduced these previous studies, the final part of this section is devoted to presenting the main novelty and contributions of this paper.

In 2012, Griffis et al. [11] focused on the distribution stage of an FSC to present an overview of CI-based optimization methods that can play a relevant role for problems like vehicle routing, supply chain risks, and disruptions. The authors emphasized how meta-heuristic techniques provide near-optimal solutions to logistics problems. Following this line of research, in 2016, Wari and Zhu [12] presented an updated survey on applying meta-heuristics to solve optimization problems in the processing (e.g., fermentation, thermal drying, and distillation) and distribution (e.g., warehousing location, production planning, and scheduling) stages of an FSC. More recently, in 2017, Kamilaris et al. [7] reviewed articles on smart farming to show how digital technologies can enhance the circularity of the FSC at the production stage. They highlighted the problems that can be approached by utilizing CI-based Statistical Learning, ANNs, and DL methods.

Complementary to the advances reported by Kamilaris et al. [7], in 2020, Sharma et al. [15] and Misra et al. [2] carried out a bibliometric analysis and a review, respectively, of CI-based Statistical Learning applications over the whole FSC. Based on their results, the authors designed a series of recommendations to design and deploy Statistical Learning-based solutions for data-driven decision-making processes in the FSC. In the same year, Camarena [10] made a critical analysis of what can be done with Artificial Intelligence, without emphasizing any single method in particular, for the transition to a sustainable FSC. Lastly, the studies of Liakos et al. [6] and Saiz-Rubio and Rovira-Mas [9], in 2018 and 2020, respectively, presented comprehensive reviews of research directed at the application of ML in the FSC production stage. The authors surveyed how ML can help farmers make more informed decisions on the management of agriculture and livestock systems.

**Figure 3:**
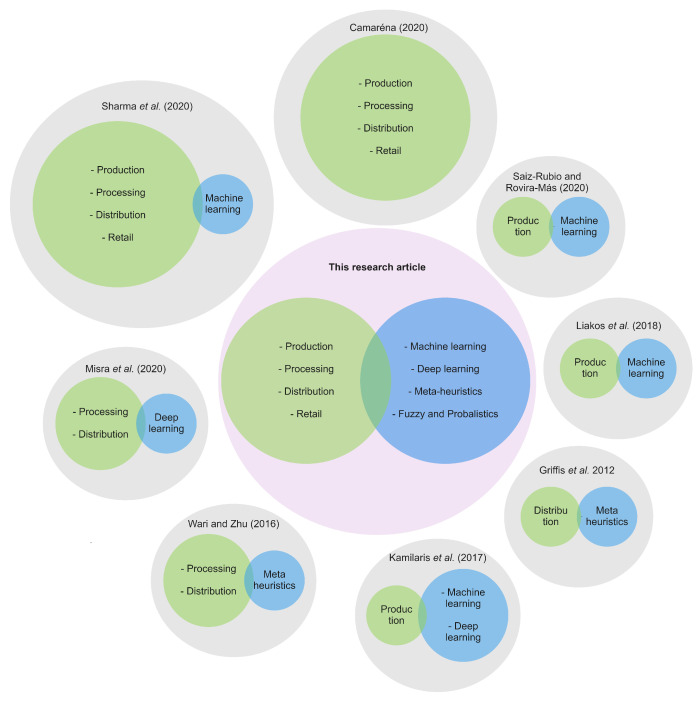
Motivations and state-of-the-art concepts at the point where FSC and CI meet.

Figure 3 presents a synthesis of the studies described above and highlights how this article complements and extends the existing literature. Each cited paper is represented by a grey circle, which can have one or two inner circles (green and blue). Green circles represent FSC stages covered by a study, while blue circles depict the CI approaches considered within it. The size of the circle is determined by the number of FSC stages and CI techniques considered in each article. Thus, a green circle would have the biggest size if the paper to which it belongs addresses the four basic stages of the FSC. The same logic is used for the blue circles: the more families of methods a paper considers, the bigger the circle’s size would be. Furthermore, we can find our research article in the center of the figure in the violet circle.

According to Figure 3 we can see that there are no research articles that present a comprehensive taxonomy at the point where FSC problems and CI converge. This means that there are no research studies that consider the problems of the four basic FSC stages, nor the diversity of the CI methods that can be applied to solve them. Instead, most of the papers focus on one or two FSC stages, and they tend to review the role a unique CI family of methods has over them. Therefore, we propose a new taxonomy that embraces the complete FSC and the five families of CI methods most commonly used in the FSC stages.

Furthermore, our proposal extends the previous classification efforts by adding a new categorization attribute, which indicates the type of FSC problem being addressed from a CI perspective. In addition to increasing the classification capacity of our taxonomy, this attribute allows us to establish a novel mapping between the FSC problems and the typologies of CI problems that can be used to approach the former ones. By doing so, we contribute to facilitating the choice of the most convenient family of CI methods to use depending on the FSC problem at hand. This represents a valuable and novel source of information for FSC researchers and practitioners who aim to incorporate CI-based solutions into their FSC applications.

## 3. A Taxonomy of CI-Based Problems in the Food Supply Chain

This section introduces details of the taxonomy proposed. First, Section 3.1 presents the methodology followed to design the taxonomy. Then, Section 3.3 and Section 3.4 show the taxonomy’s structure and describe its parts. Finally, Section 3.5 gives an overview of the categorization of FSC problems, from a CI perspective, using the taxonomy.

### 3.1. Methodology for the Design of the Taxonomy

This section introduces the methodology followed to build the taxonomy proposed. First, we note that this research paper does not aim to carry out a systematic literature review or survey. Instead, our scope lies in searching and reviewing the representative literature to propose a taxonomy that describes and categorizes FSC problems and how they are solved from a CI-based perspective. As such, the taxonomy proposed does not seek to identify all details associated with the FSC problems to maintain its comprehensibility and size. It is designed according to core characteristics that may alter the complexity and modeling of FSC problems from the CI perspective.

**Figure 4:**
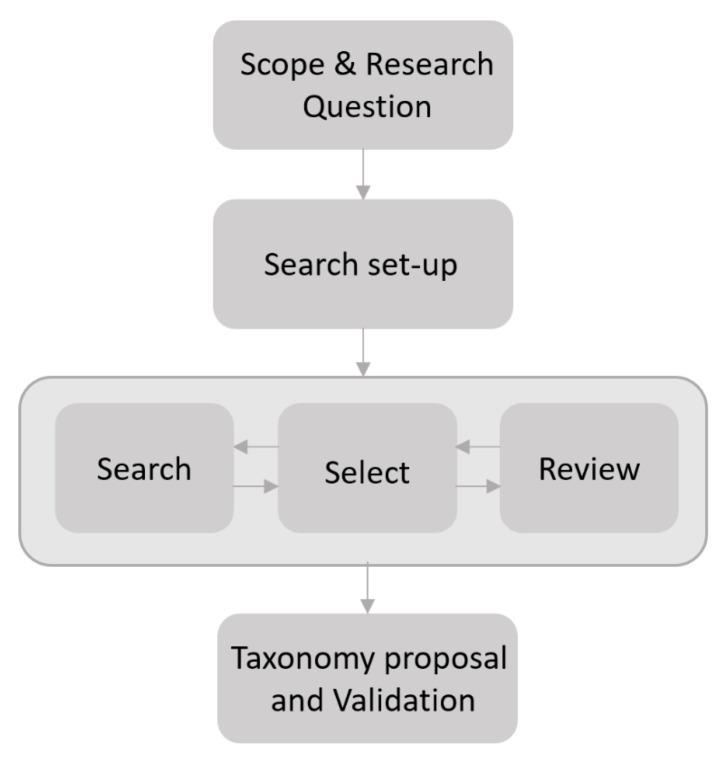
Steps followed to build the proposed taxonomy.

With these ideas in mind, Figure 4 shows the methodology followed to build the taxonomy introduced in this research paper. This methodology follows a structure-based literature review that includes the steps depicted in Figure 4. The first step is named Scope & Research Question, which aims to limit the areas of knowledge to be consulted; that is, the point where FSC and CI converge. For this step, the research questions that guided our search were: “What are the most common FSC problems reported in the literature?”, “What are the CI methods typically used to approach these problems?”, “How can FSC be categorized from a CI perspective?”, and “Is there any taxonomy to categorize FSC problems considering a CI approach?”.

The following step defined the search set-up. We define the keywords, periods, online resources, and criteria to search and review the scientific literature. The keywords considered were: Food Supply Chain(s), agrifood, fish farming, agriculture, livestock, production, processing, distribution, logistics, retail, computational intelligence machine learning, deep learning, meta-heuristics, fuzzy systems, and probabilistic methods. The period was between 2012 and 2020, and the bibliographic resources searched were the Web of Science, Scopus, and Google Scholar. Finally, the main criteria for selecting and reviewing the literature was that they be review or survey papers. The latter is based on the fact that this type of paper offers a general and consolidated overview of the state-of-the-art concerning CI-based FSC problems reported in the literature. Moreover, these types of papers allowed us to know if any taxonomy was previously proposed to classify the FSC problems.

The next stage in Figure 4 is search, select, and review. Using the search set-up mentioned above, we identified the survey and review papers introduced in the Motivation Section 2.3. Next, we analyzed the FSC problems considered in these papers, the FSC stages where the problems were located, and the families of CI methods usually considered to approach these FSC problems.

Based on the findings mentioned above, we moved to the final step of the methodology shown in Figure 4. The objective was to design a new taxonomy that embraces the complete FSC and the five families of CI methods most commonly used in the FSC stages. This taxonomy also intends to expand the previous classification efforts by adding a new categorization attribute, indicating the type of FSC problem addressed from a CI perspective. Thus, we characterized how the FSC problems identified in the previous step can be modeled from a CI perspective. To do so, we considered the typologies of problems in the CI domain (problem-solving, uncertain knowledge and reasoning, knowledge discovery and function approximation, and communication and perception) that better suited the families of CI methods considered in the studies reviewed.

Having built the taxonomy, we checked its robustness and ability to discriminate papers that approached different FSC problems. To accomplish this aim, we extracted relevant references cited by the review and survey papers previously identified, as well as the recent literature, and mapped them into the proposed taxonomy. This is shown in the following section, where the taxonomy is introduced, to validate its classification power.

### 3.2. The Taxonomy Overview

**Figure 5:**
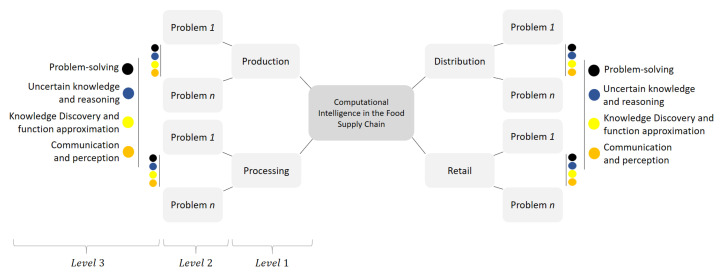
A taxonomy of computational intelligence in an FSC.

The taxonomy aims, firstly, to extend the previous classification efforts on FSC problems to cover all stages of Agrifood supply chains; and, secondly, to add a new level of categorization that allows typologies of FSC problems to be mapped to typologies of CI problems. We can see the structure of the proposed taxonomy in Figure 5. As we can see, in level one, the taxonomy includes the four basic stages of an FSC that were introduced in Section 2.2.1; that is, production, processing, distribution, and retail. Then, in level two, it contains the different categories of FSC problems that we can find in each stage. It is important to clarify that although these FSC problems have been reported previously, in related studies [2,6,7,8,9,10,11,12], to the best of our knowledge, this is the first time that their definitions are unified and consolidated in one taxonomy. Lastly, in level three, the taxonomy introduces the typologies of problems from a CI perspective. Specifically, this level seeks to classify the FSC problems depending on how they can be modeled and solved by CI methods.

Having presented the structure of the taxonomy, the following Section 3.3 details the FSC problems identified for the production, processing, distribution, and retail stages. Those problems represent the second level of the taxonomy. They are formally defined from a purely FSC perspective, and we clearly state the key objective of each problem within the particular chain stage where it is identified. Afterwards, Section 3.4 presents the third level of the taxonomy that contains the attributes to categorize the FSC problems of level 2 under a CI approach. These attributes correspond to the four typologies of CI problems that are more commonly used in the literature to address FSC problems. Finally, Section 3.5 introduces the classification and mapping of FSC problems according to how they can be modeled by CI typologies.

### 3.3. Level 2: Identification and Definition of Food Supply Chain Problems

In this section, we present the FSC problems identified for each of the FSC stages shown in Figure 5, which corresponds to the second level of our taxonomy. These problems are formally defined in more detail below.

#### 3.3.1. Production Problems

**Figure 6:**
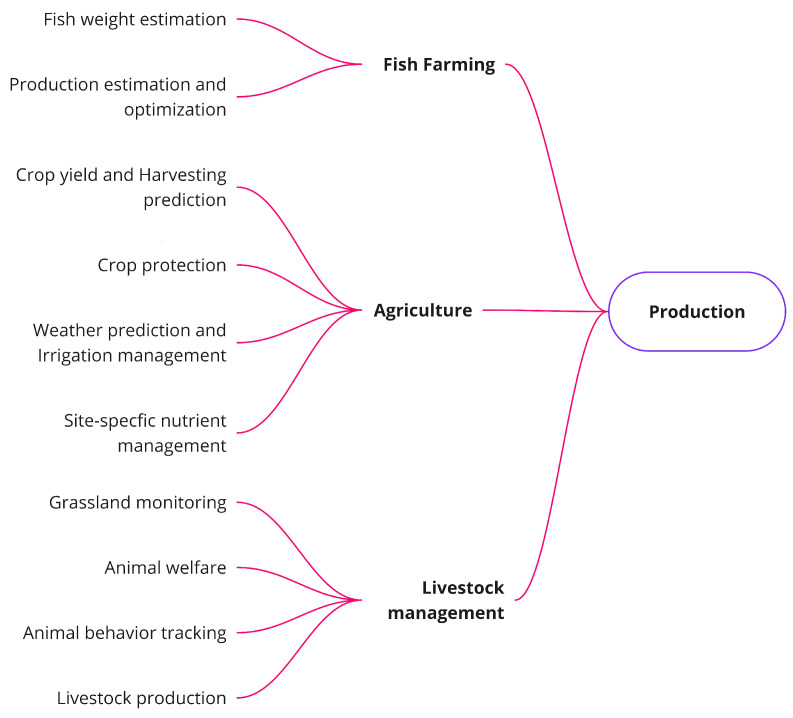
FSC problems in the production stage.

The FSC production stage can be split into three main production systems: fish farming, agriculture, and livestock. These three production systems and their associated problems can be observed in Figure 6 and they are defined below.

Fish farming (also known as pisciculture) is the production system concerned with raising fish in closed environments, such as ponds or tanks, for human consumption. Nowadays nearly half of the fish consumed in the word are raised in artificial environments [57]. Fish farming production has a high degree of complexity as it involves interrelated physical (e.g., water and nutrient supply), chemical (e.g., pH, oxygen), and environmental (e.g., waste generated) elements. Therefore, the management of this process requires advanced sensing, control, and communication technologies as well as expert knowledge to make efficient and sustainable decisions, and maximize the productivity. Within this context, the most typical CI-based processes reported in the literature are fish weight estimation [58], production estimation, and optimization [59]. Their definitions are presented below.

- **Fish weight estimation**: This process estimates fish weight considering morphological features (e.g., length, width, and mass).
- **Production estimation and optimization**: This process is centered on the optimization of fish production and forecasting of seasonal demand to adjust the production. To accomplish such aims, the optimization of production is carried out by monitoring crucial elements of fish ponds, like water oxygen levels, nutrients, and food supply, which influence the growth of fish. Meanwhile, historical records of seasonal demand are stored and continuously analyzed to determine the most suitable levels of production depending on the year and season.

The following production system considered in this study is agriculture, in particular, horticulture. Horticulture is the greenhouse industry dedicated to plant cultivation and processing of different types of crops for food and commercial consumption (e.g., flowers, fruits and nuts, vegetables and herbs). The main challenges of these production systems are to improve plant growth, yields, quality, nutritional value, and resistance to insects, diseases, and environmental stress.

In order to achieve these improvements, different processes are managed to try and maintain a balance between efficiency, productivity, and sustainability, such as monitoring and controlling indoor-outdoor climate conditions, crop management, and production forecasting, among others. They are commonly approached in the specialized literature [6,9,60] in open-field agriculture and intensive horticulture. Within the most representative processes, we find the crop yield and harvest forecasting [60,61,62], crop protection [63,64], weather prediction and irrigation management [65,66], and site-specific nutrient management [67,68]. These processes, which are shown in Figure 6, are defined as follows.

- **Crop yield and harvesting prediction**: This problem is focused on yield estimation to match crop supply with demand and on crop management to increase productivity.
- **Crop protection**: This is based on the identification and diagnosis of biotic (infestations, diseases, and weeds) and abiotic (nutrients, water deficiency) stress factors that affect crop productivity.
- **Weather prediction and irrigation management**: This problem is mainly concerned with weather forecasting for the optimal use of water, which enables the design and deployment of crop irrigation scheduling and planning.
- **Site-specific nutrient management**: This is based on the management of soil quality to determine which nutrients need to be supplied in order to maintain the chemical characteristics required for the crop under consideration.

Lastly, the third production system considered for the production stage is livestock. This production system is dedicated to the growth and management of domestic animals (e.g., cattle, sheep, and goats) raised in agricultural settings to generate food products like meat, eggs, or milk, among others. Livestock can be carried out through either extensive or intensive systems. Extensive systems involve animals roaming grasslands (usually under the supervision of a herdsman). Diversely, intensive livestock is located in closed infrastructures and is equipped with ICT technology, which enables animals to be monitored in real-time. Within these production systems the most typical problems we come across are grassland monitoring [69], animal welfare [70], animal behavior tracking [71], and livestock production prediction and optimization [72,73], as shown in Figure 6. The formal definitions of these problems from an FSC perspective are listed below.

- **Grassland monitoring**: This problem is related to the accurate identification of grassland inventories in order to discriminate between the most suitable types for livestock purposes.
- **Animal welfare**: This is focused on the pattern classification of ingestive behavior in grazing animals for studies of animal nutrition, growth, and health.
- **Animal behavior tracking**: This is based on the use of behavior analysis to detect early signs of health issues and promote early intervention.
- **Livestock production**: This problem is centered on predictions and estimations of farming parameters to optimize the economic-energy efficiency and sustainability of production systems.

#### 3.3.2. Processing Problems

**Figure 7:**
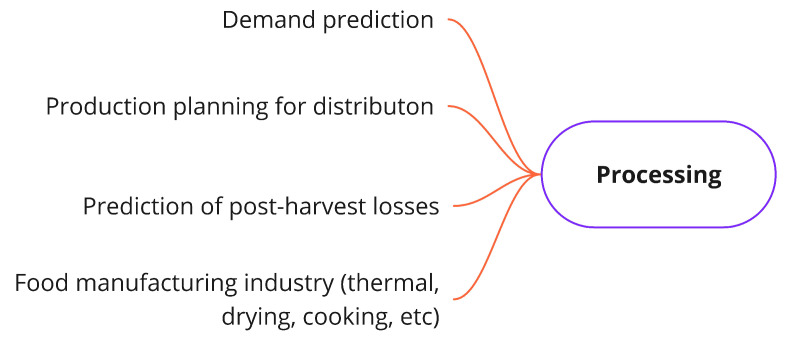
FSC problems in the processing stage.

Once the raw materials of food products have been grown, they are delivered to the next step of the FSC, or ‘processing’. Different industrial processes (e.g., washing, disinfecting, packaging) are carried out in this stage to transform the raw output of production into edible food products. Depending on the production system under consideration and the food products obtained from them, diverse industrial processes can be followed to obtain the goods that move on to the distribution stage. However, in spite of such production particularities, we have identified a set of common problems that could occur in the three production systems presented in the section above. These problems are shown in Figure 7 and they are: demand prediction [74], production planning for distribution [75], prediction of post-harvest losses [76], and manufacturing industry processes, such as cooking, drying, and others [77].

- **Demand prediction**: This problem in concerneed with the demand prediction of food requirements to avoid overstocking, overproduction, and over-utilization of resources. The key idea is to estimate the quantity of food products that will be sold to define how much raw material needs to be processed.
- **Production planning for distribution**: This is centered on production planning to match distribution requirements. This problem is mostly determined by the sale volumes that a particular food product is expected to have.
- **Prediction of post-harvest losses**: This is focused on making estimations of food losses associated with the processing procedures carried out after harvesting raw materials coming from the production stage.
- **Food manufacturing industry**: This is associated with the optimization of the processing technologies required to transform raw foods into edible food (e.g., thermal, drying, contact cooking, microwave heating, etc.). These processes are performed using industrial machinery.

#### 3.3.3. Distribution Problems

In the third step of the food supply chain, food products ready for human consumption are received from the processing stage to be delivered to end-consumers. Specifically, finished products arrive at warehouses, and from there, the shipment department is in charge of defining the most suitable strategy to deliver products to end-consumers. The essential purpose is to distribute food products on time by the date specified in the retail stage.

**Figure 8:**
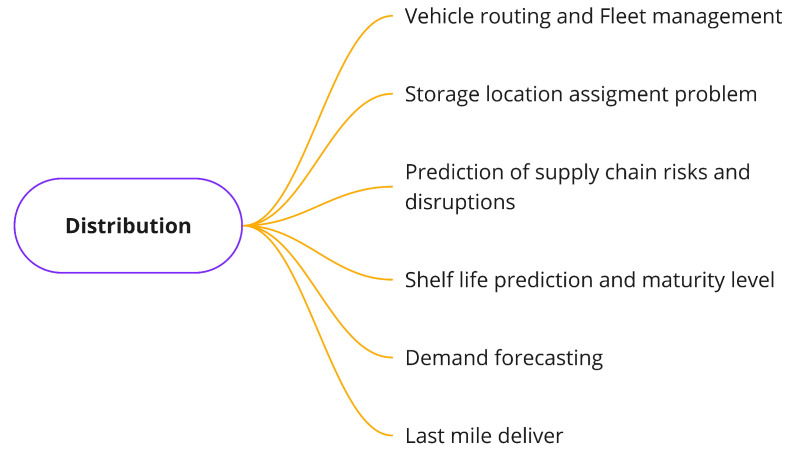
FSC problems in the distribution stage.

For this particular stage of the FSC, the most common problems reported in the specialized literature are shown in Figure 8 and defined below. These problems include vehicle routing and fleet management [78,79], storage location assignment [80,81], prediction of supply chain risks and disruptions [82,83], shelf life prediction and maturity level [84,85,86], demand forecasting [87], and last mile delivery [88].

- **Vehicle routing and fleet management**: This is focused on determining the most optimal route for the delivery of food under different scenario constraints (e.g., size of the fleet, fuel availability, etc.).
- **Storage location assignment problem**: This problem is concerned with deciding the most suitable way to store food products in a set of warehouses in order to cope with daily demand operations.
- **Prediction of supply chain risks and disruptions**: This is concerned with the forecasting of potential disruptions in the logistics of food products and their associated food losses.
- **Shelf life prediction and maturity level**: This problem is related to the forecasting of shelf life based on data sensed during the distribution process.
- **Demand forecasting**: This consists of understanding demand behaviors and forecasting user demand generated from the retail stage. Thus, it is possible to optimize the delivery routes and warehouse locations used during the distribution stage.
- **Last mile delivery**: This problem is dedicated to the delivery of food products using the local road transport network (last mile) of cities.

#### 3.3.4. Retail Problems

**Figure 9:**
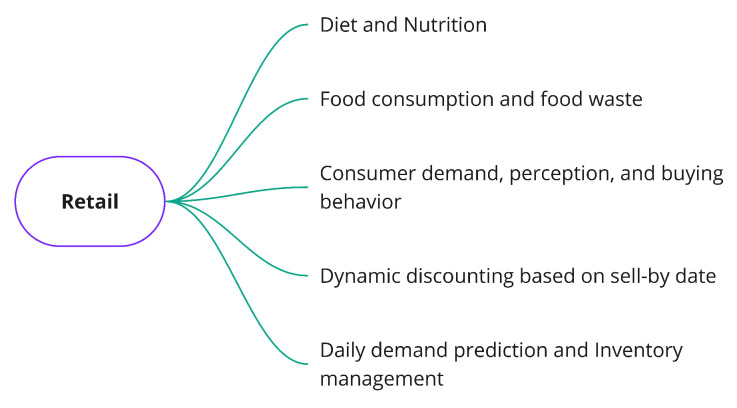
FSC problems in the retail stage.

In the final part of the FSC, we find the retail stage. At this point, food products are received through the distribution channels, ready for sale. This stage encompasses the concept of an ’end-consumer’, which could be supermarkets or clients that go to these places to buy food products. The most common problems identified in the literature for this stage of the supply chain are defined below and are also summarized in Figure 9.

Lastly, we characterized the retail stage (Figure 5). Retail-related problems commonly addressed with CI, in this link of the FSC, are diet and nutrition applications [89,90], food consumption and food waste [91,92], consumer demand, perception and buying behavior [93,94], dynamic discounting based on the sell-by date [95], and day demand prediction and inventory management [74].

- **Diet and Nutrition**: This is based on estimating nutrient values using the classification of food dishes and dietary assessment.
- **Food consumption and food waste**: This problem is associated with the identification and prediction of food waste based on the buying and storage behavior of end-customers.
- **Consumer demand, perception, and buying behavior**: This problem is focused on determining consumer profiles in order to predict buying behaviors and support the management of shop counters.
- **Dynamic discounting based on sell-by date**: This is centered on automated price changes at supermarkets based on the sell-by date. The objective is to offer larger discounts for items with the shortest remaining shelf life.
- **Daily demand prediction and inventory management**: This problem consists of predicting daily demand to better manage product stocks at supermarkets.

### 3.4. Level 3: Typologies of CI Problems

In this section, we introduce the attributes that represent the most typical typologies of CI modeling approaches used in the FSC literature. The attributes correspond to the third level of the taxonomy shown in Figure 5, and they are presented below.

- **Problem-solving**: This category is related to problems of complex decision-making processes that need to be solved, keeping two key objectives in mind: quality of the solution and the computational time required to solve it. As a common denominator, this attribute categorizes problems that are *NP*-hard. Thus, this class embodies FSC problems for which there is no certainty that the method can optimally solve them in a polynomial time (time complexity [96]) with respect to the input data size. This category includes, for example, optimization or search problems such as the vehicle routing problem in the transportation stage of the FSC.
- **Uncertain knowledge and reasoning**: This category corresponds to FSC problems characterized by having partially observable, non-deterministic, vague, or imprecise data. In such uncertain scenarios, this attribute represents problems that can be addressed in two possible ways. First, by using an approach that acts based on assumptions of uncertain input data in order to give a probabilistic-based solution to the problem at hand. Or second, by representing and reasoning with the partially available information in a manner similar to the way that humans express knowledge and summarize data. This second approach allows non-exact data to be represented in linguist terms in order to make decisions within certain margins of correctness.
- **Knowledge discovery and function approximation**: This class represents FSC problems that are distinguished by having large volumes of data, which enable understanding and useful knowledge to be extracted from them. Such knowledge could be used to make either predictions of future events or discrimination and recognition of patterns. These types of problems can usually be addressed with methods that can be trained using the available data to learn a specific task.
- **Communication and perception**: This category consists of FSC problems focused on the automatic extraction, analysis, and understanding of information obtained from digital images, texts, or voice recordings. It is worth noting that within the FSC domain, most problems solved using a communication and perception approach are focused on designing and developing autonomous computer vision systems. These systems are able to process high-dimensional data to support decision-making; from object detection to video tracking and object recognition.

### 3.5. Mapping Process between Level 2 and Level 3: Classification of FSC Problems from a CI Perspective

This section presents the categorization of FSC problems previously identified in Section 3.3 from a CI perspective. Specifically, every problem is categorized according to the four typologies of CI problems that we described above: problem-solving, uncertain knowledge and reasoning, knowledge discovery and function approximation, and communication and perception. In addition, using relevant work from the recent FSC literature, we show how the taxonomy is able to effectively and robustly discriminate these papers. Thus, Section 3.5.1 is devoted to classifying the FSC problems coming from the production stage. Then, Section 3.5.2 introduces the classification of problems defined for the processing stage. Lastly, Section 3.5.3 and Section 3.5.4 expose the categorization of problems for the distribution and retail phases, respectively.

#### 3.5.1. Classification of Production Problems

**Figure 10:**
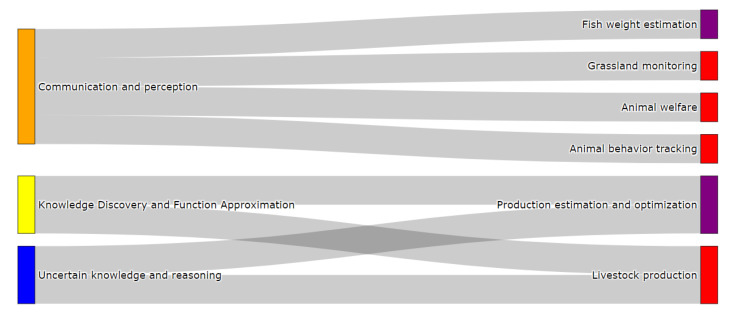
Fish farming and livestock problems classified by the proposed taxonomy.

Figure 10 presents the categorization of production problems for the fish farming and livestock cases. The attributes of the taxonomy are depicted on the left in Figure 10, whereas the fish farming (in purple) and livestock (in red) problems are found on the right. The problems and attributes are connected by gray bars that connect the ways in which a specific problem can be modeled from a CI perspective. Fish weight estimation, grassland monitoring, animal welfare, and animal behavior tracking are problems that are classified using the communication and perception attribute. This is justified due to the fact that these problems are usually characterized by having image and video records as input data (non-structured data). Having such data implies the use of automatic information extraction by means of computer vision systems, based on DL, which allow extraction of high-dimensional patterns embedded in image and video data [97,98,99,100,101,102,103].

The production estimation and optimization and livestock production problems in Figure 10 are classified using the knowledge discovery and function approximation and uncertain knowledge and reasoning attributes. Such categorization is determined by the central characteristics of these problems, which are concerned with the prediction and optimization of production values using historical data records (structured data) [104,105]. Besides, production forecasting contains uncertainties regarding external factors of the production systems, which encourage the use of probabilistic methods, such as the studies carried out by [106,107].

**Figure 11:**
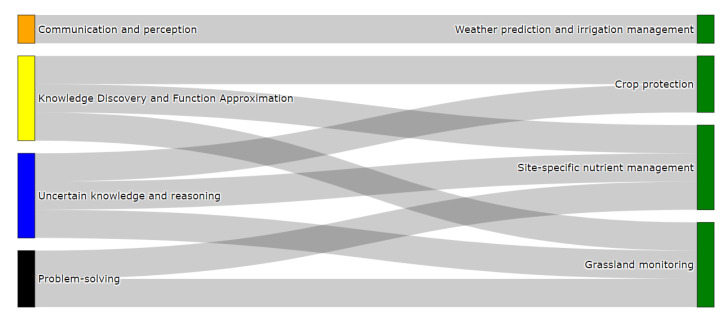
Agriculture problems classified by the proposed taxonomy.

Figure 11 shows the categorization of agriculture problems. As we can seen, crop protection, which usually involves video and image records, is categorized as a communication and perception problem commonly used in DL methods like convolutional and recurrent neural networks [108,109,110]. On the other hand, the remaining agricultural problems can be categorized under knowledge discovery and function approximation, problem-solving, and uncertain knowledge and reasoning attributes as explained below. In terms of knowledge discovery and function approximation, this attribute categorizes problems focused on the prediction of greenhouse conditions for the optimal management of crop production, nutrients, and irrigation supply. This can be done using supervised learning techniques that forecast variables of interest like temperature, water, or nitrogen [6,111]. Secondly, the problem-solving attribute categorizes the weather prediction and irrigation management and the site-specific management problems. These problems can be defined as optimization scenarios in which we can find concrete values associated with weather, irrigation, and nutrients. In this case, the most common approach to solving them is based on meta-heuristics [112,113,114].

Finally, the uncertain knowledge and reasoning attributes characterize the same problems that fall into the knowledge discovery and function approximation attribute. The difference lies in the fact that the agriculture problems are approached as decision systems under uncertainty. In this case, fuzzy logic is the most suitable strategy to accomplish such an aim [115,116,117] Specifically, the fuzzy approach allows us to evaluate whether particular actions need to be taken according to sensed and predicted conditions coming from agriculture systems (e.g., optimal management strategies for the control of temperature control inside greenhouse systems).

#### 3.5.2. Classification of Processing Problems

**Figure 12:**
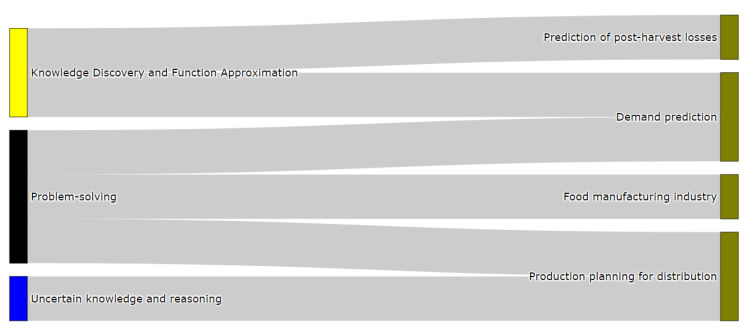
Processing problems classified by the proposed taxonomy.

Having presented the production stage, Figure 12 features the categorization of problems in the processing stage. Any of the depicted FSC problems are classified using the communication and perception attribute, mainly because the type of input data they handle is not associated with images or video records. In contrast, when the aim is to determine future scenarios based on the available data, all problems tend to have historical data as their input data.

Precisely, the knowledge discovery and function approximation attribute categorizes the prediction of post-harvest losses and demand prediction problems as being supervised learning problems, whose aims are to claasify and forecast the objective variable. For instance, we consider research from [8,118] to be within this category. Following the taxonomy’s attributes in Figure 12, the problem-solving attribute can represent and model demand predictions, the food manufacturing industry, and production planning for distribution problems. In this scenario, they are classified as being optimization problems, with the objective of optimizing procedures related to the problems listed. An example, for instance, could be the optimization of industrial manufacturing processes, like heating and drying, using meta-heuristic techniques. Examples of studies categorized by the taxonomy of the previously mentioned approaches are found in [119,120].

Lastly, the uncertain knowledge and reasoning attribute system also classifies production planning for distribution problems. Probabilistic methods tend to be used to approach these kind of problems as production planning can incorporate uncertainty coming from processes that are not directly related to the production process, which can cope with probability theory [121]. An example of this last situation could be a change in the delivery date due to delays attributable to the weather [122].

#### 3.5.3. Classification of Distribution Problems

**Figure 13:**
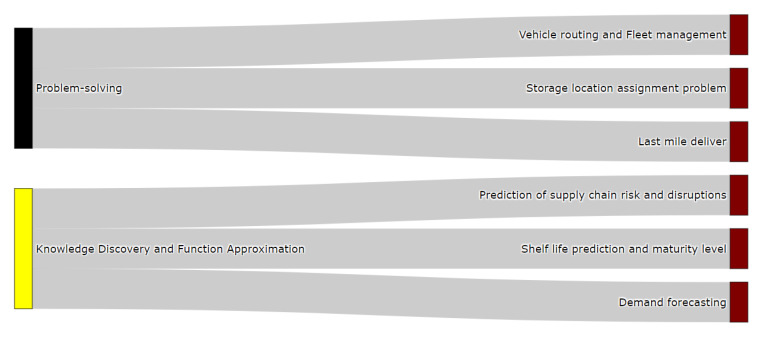
Distribution problems classified by the proposed taxonomy.

Figure 13 exhibits the classification of FSC problems in the distribution stage. Unlike the two previous stages (production and processing), the predominant approaches here are the ones imposed by the problem-solving and knowledge discovery and function approximation attributes. The former attribute categorizes vehicle routing and fleet management, the storage location assignment problem, and last mile delivery problems, as they are devoted to optimizing routing and delivery situations. The purpose of these applications is to optimize, using meta-heuristics, a concrete goal under different constraints; for instance, fleet size, available fuel, and others. Representative studies that fall in this category are [123,124,125,126,127].

The knowledge discovery and function approximation attribute includes prediction of chain and disruptions, a shelf life prediction and maturity level, and demand forecasting problems. This attribute classifies these problems under a supervised learning perspective, where the aim is to predict expected values, such as we can see in research carried out in [84,128,129,130,131]. For instance, potential disruptions to the cold food products chain, or an estimation of how much product volume needs to be distributed to meet retail demands.

#### 3.5.4. Classification of Retail Problems

**Figure 14:**
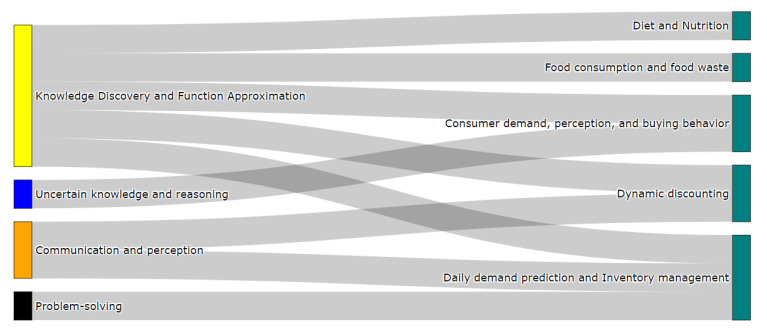
Retail problems classified by the proposed taxonomy.

Finally, Figure 14 introduces the classification of problems in the retail stage of the FSC. In this last step, the communication and perception attribute appears once again to represent the problems in which the input data correspond to non-structured data, such as images (dynamic discounting, daily demand prediction, and inventory management) [95,132,133,134,135]. For these particular cases, the problems can be modeled using DL techniques to determining price discounts based on stock levels inside supermarkets and by managing inventories according to food product existence.

Contrarily, the knowledge discovery and function approximation attribute includes problems associated with the extraction of patterns (food consumption and food waste), the prediction of future values related to consumer demand and buying behavior, and the generation of healthy menus or estimating nutritional values. Research articles on this attribute include [89,90,136,137,138,139]. Furthermore, this attribute can also classify the dynamic discounting and daily demand prediction and inventory management problems when their input data corresponds to structured information like historical records.

In addition to the attributes mentioned above, the uncertain knowledge and reasoning, and problem-solving attributes can be used to categorize a couple of problems in the retail stage. Those problems are consumer demand, perception, and buying behavior, as well as daily demand prediction and inventory management. Consumer demand, perception, and buying behavior can be approached with a probabilistic system [140,141,142], for instance, uncertainty concerning what food products are expected to be bought. Meanwhile, daily demand prediction and inventory management can be addressed with an optimization paradigm [143,144]. For this case, the aim is to optimize stock levels in such a way that food waste can be decreased or even to avoiding over-stocking issues completely.

## 4. Guidelines for the Use of Computational Intelligence Approaches in the Food Supply Chain

**Figure 15:**
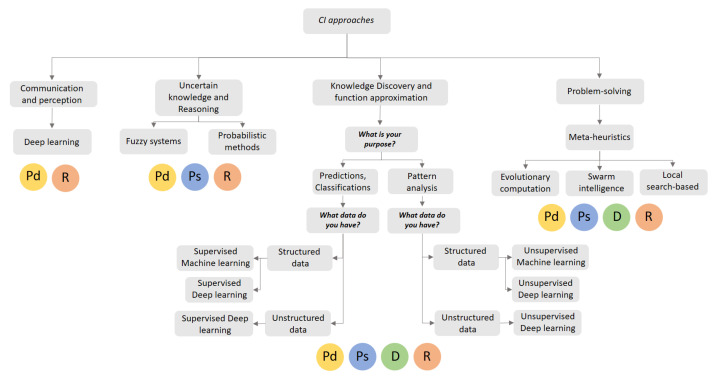
Guidelines for the method selection problem in the food supply chain. Pd: production, Ps: processing, D: distribution, R: retail.

Having presented and validated the taxonomy of FSC problems, this section presents a set of guidelines for researchers and practitioners in FSC for the use of CI within this domain (Figure 15). Concretely, we try to guide the users to (1) select the typology of a CI problem that they are addressing; and (2) identify what families of CI methods could be more suitable for the problem at hand. The latter does not mean that in all cases the family of methods suggested is the most appropriate, as this may depend on the specific characteristics of the problem being addressed.

The guidelines depicted in Figure 15 start with a basic question posed to the user: “What is the purpose and modeling characteristics of the problem at hand?” (it could be communication and perception, uncertain knowledge and reasoning, knowledge discovery and function approximation, and problem-solving). If the purpose is the automatic analysis and extraction of information from digital images to decide on the action to be taken regarding the management of food supply systems (communication and perception), the suitable family of methods would be deep neural networks (e.g., convolutional neural networks). This family of CI methods enables the creation of computer vision systems, which allows the environment of object characteristics to be perceived in a visual way. Based on this visual analysis, these systems communicate or recommend actions that achieve desired states or match predefined conditions (e.g., identify the quality of potatoes in order to evaluate the units that are either damaged or edible).

If the objective of the user is to handle problems characterized by partially observable, non-deterministic, or imprecise data (uncertain knowledge and reasoning), fuzzy systems or probabilistic methods are recommended. For the former CI approach, it is important to highlight that fuzzy systems need to be paired with hardware (e.g., PID controllers) to work properly in food applications. This is due to the fact that hardware components allow decisions made by fuzzy systems to be translated into actions (e.g., management of nutrients and irrigation supply inside a greenhouse system depending on conditions associated with temperature). Probabilistic methods are suitable for making estimations of relevant variables (e.g., planning production according to seasonal demand) in scenarios with partially observable data.

When the users’ aim is directed at making predictions from historical data, making classifications that discriminate between data categories, or finding hidden patterns in data, the best modeling approach to use is knowledge discovery and function approximation. Firstly, for predictions and classifications, the user should determine the type of input data at hand. In general terms, the input data can be structured (e.g., historical records, tabular data) or unstructured (e.g., video, images). In the former, and depending on the data size, the supervised learning techniques are the CI methods to be used when facing small, medium, and large data no bigger than 40–50 gigabytes. Supervised DL, however, is the recommended approach for big datasets. In terms of making predictions and classifications when using unstructured data, supervised DL would be the most suitable learning approach; while unsupervised ML or unsupervised DL are the recommended CI approaches for pattern analysis. Finally, as we can see in Figure 15, the other category of problems that users might face is problem-solving. In this case, the user’s aim is to optimize particular values in order to achieve a desired level of performance. As such, the above-suggested approaches are therefore all meta-heuristics (e.g., EC, SI, and local search-based techniques).

In addition to the analyses presented above, the bottom part of Figure 15 also depicts which FSC stages the four CI modeling approaches (and their associated methods) are usually applied in. Fuzzy systems and probabilistic approaches are typically considered for control applications in the production, processing, and retail stages. In contrast, optimization with meta-heuristics and prediction-classification-pattern analysis with ML and DL are modeling perspectives that are considered in the entire FSC process. The contributions of communication and perception approaches using DL methods tend to be more typically focused on the production and retail stages.

## 5. Conclusions

This final section introduces the main reflections drawn from the research carried out in this paper. Section 5.1 introduces the summary and conclusions. Then, Section 5.2 details a set of challenges and research opportunities to encourage further exploration and use of the possible contributions that CI might bring to the FSC field.

### 5.1. Summary

This paper has proposed a new and comprehensive taxonomy of FSC problems under a CI paradigm for three representative supply chains: agriculture, fish farming, and livestock. The taxonomy was built based on three levels in order to categorize FSC problems according to how they can be modeled using CI approaches. The first and second levels are focused on identifying the chain stage (production, processing, distribution, and retail) and the specific FSC problem to be addressed (e.g., vehicle routing problems in the distribution stage). The third level presents the typologies of FSC problems from a CI perspective, and aims to categorize FSC problems depending on how they can be modeled and solved by CI methods. In the third level of the taxonomy we have defined four attributes, presented as follows, (1) problem solving, which is in charge of classifying FSC problems focused on optimizing processes; (2) uncertain knowledge and reasoning, which concers problems that have partially observable, non-deterministic, incomplete, or imprecise data; (3) knowledge discovery and function approximation, which has the role of categorizing problems that aim to make predictions of future scenarios, classification of variables, or analysis of patterns embedded in data; (4) communication and perception, groups FSC problems that involve computer vision systems to sensing and suggesting plausible actions to take in order to intervene in such environments.

To check the robustness of the taxonomy, we categorized FSC problems with CI methods, especially in the production, processing, distribution, and retail stages. Here, it is relevant to highlight that we introduced a set of unified definitions for these problems. As a result, we were able to draw some interesting conclusions. In the fish and livestock cases of the production stage, using the DL and the communication and perception attribute significantly influences applications (e.g., fish weight estimation, grassland monitoring, animal welfare) where the input data is determined by image and video records (non-structured data). In contrast, we have the case of classic ML, which is narrowed to FSC problems, and for which, the objective is to make production predictions using historical data records (structured data). In the case of agriculture production systems, the scope of the CI approach is broader. Specifically, we found that DL, ML, FL, and Meta-heuristics are methods for modeling production problems related to crop protection and yield, weather prediction, and irrigation and nutrient management.

In the processing stage, ML, meta-heuristics, and probabilistic methods are the CI approaches commonly used. In terms of ML, the aim is to extract patterns and forecast objective variables like demand prediction and prediction of post-harvest losses. As for meta-heuristics and probabilistic techniques, they aim to optimize food manufacturing processes (e.g., heating, drying) and production planning for distribution. Further down in the supply chain, the predominant family of CI methods is meta-heuristics, found in the distribution link. This data-driven approach is devoted to optimizing the routing and delivery problems under various constraints such as fleet size and available fuel. Lastly, DL is the principal CI approach in problems with non-structured input data (e.g., dynamic discounting, diet, and nutrition) in the retail stage. Classical ML has been used to extract patterns (food consumption and food waste) and predict consumer demand and buying behavior.

The taxonomy allowed us to figure out which modeling approaches are more typically considered when dealing with problems at the four supply chain stages. In this manner, we gave a general overview of well-established tendencies regarding CI across the three supply chains considered. Thus, the definition and classification of FSC problems helped us introduce guidelines for the incorporation and use of CI in the food industry. These guidelines are built upon CI’s primary purposes in the food supply chain: communication and perception, uncertain knowledge and reasoning representations, knowledge discovery and function approximation, and problem-solving. These guidelines aim to help non-expert CI users to identify families of methods that can supply a solution for their particular CI-based needs in different FSC problems.

In summary, the taxonomy analysis suggests that there is no family of CI methods that best suits all FSC problems. However, we state the need for a comparison framework that allows the description and analysis of the performance of different CI methods in diverse supply chain problems. In this context, the taxonomy presented sets up the basis for a common framework that, in further research, will facilitate experimentation in order to determine which CI approaches are more appropriate for each type of FSC problem. This may also help determine a suitable baseline of methods to make fair comparisons, depending on the family of CI methods chosen for the FSC problem at hand.

### 5.2. Challenges and Research Opportunities

As industry 4.0 is flourishing for the FSC management and operation, emerging research paths arise for CI to yield more robust, interoperable, and accurate methods [145,146]. Therefore, this section points out challenges and research opportunities that the community should explore to enhance the contributions that CI can bring to the digitization of the FSC. These challenges are motivated by the gaps located at the intersection of FSC and CI, which were identified through the proposed taxonomy.

#### 5.2.1. Data Fusion from Different Data Sources

Significantly, few CI methods can incorporate data from different types of sources. Besides, in real scenarios, the data available from a unique type of sensor might not be sufficient to fully represent the FSC problem that is intended to be addressed. For instance, different Internet of Things (IoT) devices (e.g., agricultural environment monitoring systems, GPS, cameras) provide diverse data for the optimum management of production systems [147,148,149]. Amongst the relevant data for the aforementioned example, we find temperature, humidity, localization coordinates, crop images, and others.

As a common denominator, all the data associated with the aforementioned variables can have a different format, ranging from historical records in the form of tables, to non-structured data like images. Additionally, all of them offer valuable information for the purpose at hand (e.g., prediction of crop production or management of pest diseases). In this context, the challenge lies in defining guidelines for the harmonizing and fusion of data from diverse sources. Such guidelines should consider that each FSC stage can add particularities to the data for the CI-based problem under consideration. How to properly collect and generate a single dataset with information obtained from varied and different sources, which are fed into a CI method, is a research opportunity that must be addressed to further enhance CI contributions in the FSC domain.

If the integration process is not done correctly, inconsistencies will appear, resulting in a decrease in the performance of CI approaches [150]. Hence, merging data from different input sources presents a notorious problem that commonly attracts more issues, such as inconsistent, duplicate, redundant, and correlated data. One potential research direction to take to help cope with this challenge could be designing automatic preprocessing approaches that fuse and harmonize data sources to provide the accepted input format of CI methods. For the latter, it is important to note that every CI approach requires different input data formats, which could split the design of the aforementioned automatic data preprocessing methods into diverse paths depending on the particular family of CI methods under consideration.

#### 5.2.2. Real-Time Data and Incremental Learning

In supervised learning, the input data is available before starting any training processes. Here, the task is to build up a model from that data using a batch approach. The latter means that DL and ML methods use all available samples in the input data to build and train a model to make predictions or classifications when new data comes into the trained model. Currently, most DL and ML applications are focused on the batch learning approach, wherein data are given before training the ML models [151]. In this context, model training and optimization processes are purely based on the aforementioned input dataset, whose data distribution is supposed to be static. Nevertheless, such a static approach is not the case for real CI-based applications within the FSC.

DL and ML methods must real FSC scenarios, wherein different IoT devices continuously generate new data streams. For instance, dynamic discounts in the retail stage or the management of greenhouse systems whose conditions must be constantly monitored to guarantee the optimal control of crops are examples of real-time data streams. Therefore, the key challenge is to design ML and DL methods that adapt to real-time data, and work with limited resources (e.g., memory), while maintaining their predictive capacities.

Further research is needed to deal with the aforementioned challenge, and should include the concepts of incremental learning [152,153] in the design and deployment of DL and ML methods in FSC problems. Furthermore, although incremental learning is a suitable strategy when dealing with the adaptation of DL and ML to real-time data streams, the concept of incremental learning brings up other issues that must be considered when it is included in the FSC context. These issues are introduced in more detail below.

The first issue is related to the concept of drift, which is associated with changes in the new input data’s statistical properties (e.g., statistical distribution), which can highly influence the performance of pre-trained DL or ML methods [154]. Secondly, we have the stability-plasticity dilemma that, in noisy environments, refers to when and how to adapt the current model to the new data stream [155]. On the one hand, quick updates enable fast adaptations of the model according to new data; however, at the same time, old information that could be useful later in the process is forgotten just as quickly. On the other hand, slow adaptations can be made, and old data is retained longer, causing the model’s reactivity capacity to decrease; thereby, affecting the model’s forecasting power. Thus, defining policies to balance the two scenarios mentioned above is a relevant challenge to support the inclusion of CI approaches in real-time data environments.

Finally, the third challenge associated with incremental learning is adaptive model complexity [156]. This issue is concerned with the fact that as concept drift events become more frequent, increasing model complexity is required. Although the latter improves the model’s performance, increasing its complexity leads to an increase in the need of more computational resources, which are not always affordable or available in different domains, such as FSC.

#### 5.2.3. Explainability of Computational Intelligence Methods

Nowadays, CI systems have increased their capacity to carry out different tasks (e.g., predictions, classification) with almost no human intervention and are achieving high proficiency levels. In a narrow scope, if the only objective when evaluating CI methods is performance, such an object has clearly been achieved thanks to this increasing capacity. Nonetheless, when decisions derived from CI-based techniques affect the dynamics of human life (e.g., social, cultural, environmental dimensions), understanding how CI systems have achieved this performance becomes necessary [157].

While some CI approaches, such as fuzzy logic and ML methods (e.g., rule-based learners, decision trees), can provide different degrees of interpretability and transparency of decisions made in their inner structure; other methods cannot provide this information (e.g., black-box decision systems [158]). In these cases, there are no clues regarding the systems’ decisions to obtain the final result. One example is DL methods, whose complex inner structure is full of intricate connections between layers and neurons and have a broad space of hyperparameters. Therefore, understanding where the high performance of DL methods comes from is quite tricky.

As DL is increasingly being employed in diverse FSC applications due to its performance capacities, the demand for interpretability, explainability, and transparency is growing between diverse FSC stakeholders. Such demand lies in the fact that decisions made based on CI systems can lead to actions, which can not be justified or that require detailed explanations [159] in order to enhance the management of the supply chain. For example, the shelf-life of perishable products needs to be predicted in the distribution stage. However, understanding the factors that influence the deterioration or preservation of food products is vital to improve the management of distribution chains. The latter is known as explainable artificial intelligence (XAI) and is widely acknowledged as a crucial concept to be taken into account in the practical deployment of CI methods [160].

In this sense, as stated by [160], the challenge of XAI in different knowledge domains, such as FSCs, is creating more explainable methods while maintaining their output performance. Besides, it is crucial to facilitate stakeholder understanding and trust in decisions supported by CI systems whose outputs become the basis for different actions along the whole supply chain.

#### 5.2.4. The Method Selection Problem

As shown in this research paper, depending on the FSC problem at hand, different types of CI methods could be used. In this sense, Section 4 provides helpful information to guide non-expert CI users when choosing the most suitable CI family of methods depending on the purpose to be pursued (e.g., prediction, classification, optimization). In this context, keeping in mind the no-free-lunch theorem [161] (which states that there is no unique algorithm that can be competitive in all problems), the proposed guidelines contribute to dealing with the model selection problem in the FSC area. Nevertheless, having identified the most suitable families of CI methods, it is still difficult to choose the best method within each family.

For instance, if the objective is to optimize a food production process in the processing stage, the critical question would be to ask what meta-heuristic approach is the most suitable for the considered problem. The answer to this question could be to use an EC, SI, or local-based approach. However, solving such a decision requires a thorough experimentation that demands a large amount of effort and is time-consuming [162]. Another similar example of the method selection problem is the case of ML. FSC data allows different variables of interest to be predicted under diverse scenarios, ranging from fish farming production to agriculture. These characteristics of the production context influence ML method performance, and selecting the most appropriate method from a pool of candidates is a time-consuming task. Thus, the automation of choosing the best CI technique from a predefined set of methods of the same family is a crucial challenge that could help increase inclusion of CI into the FSC.

Currently, there are well-established automated approaches focused on ML and DL. Therefore, we can use the automation of complete ML or DL workflows, known as Automated Machine learning (AutoML) [163] and Automated Deep learning (AutoDL) [164]. Such approaches are considered to be promising strategies to reduce human effort and time cost of ML and DL in research areas in which specialized knowledge is an asset that is not always available or affordable. Particularly, AutoML and AutoDL seek to find competitive ML pipelines (the workflow from data preprocessing to model selection) and neural network architectures automatically without almost any human intervention. The central purpose is maximizing or minimizing a performance metric on input data without it having to be specialized in the problem domain where the data comes.

AutoML and AutoDL methods have been successfully used in other areas [165,166]. However, an extensive analysis to determine their strengths and weaknesses has not been carried out in very diverse learning tasks, such as FSCs. Additionally, the concept of AutoML and AutoDL could be extrapolated to an even broader space, such as automated computational intelligence. In this case, the focus would be on supporting non-expert users in selecting other CI techniques like meta-heuristics and its sub-families (e.g., EC, SI, local-based approaches). In this context, the research challenge lies in the design and development of automated methods for other families of CI methods, including fuzzy logic, meta-heuristic, probabilistic methods, and others.

#### 5.2.5. Interoperability and Deployment of CI with Information and Communication Technologies

CI is only one part of the entire ICT ecosystem required to digitize processes in the FSC. IoT technologies, information storage and management technologies, data analytic and visualization tools, or decision support technologies are other relevant components without which CI cannot reach its full potential. All these technologies have switched the focus from traditional automation of FSC processes to a cloud-based paradigm where all ICTs operate efficiently and intuitively [167]. Within the cloud-based approaches, major technologies must be able to interoperate with each other and themselves. Thus, such interconnectivity and exchange of information are some of the main challenges raised when incorporating CI and other industry 4.0 approaches into the daily operation and management of FSCs [168].

One of the latent issues is the lack of standardization of the data models with CI methods. Ultimately the goal of Industry 4.0 in FSCs is to incorporate semantic interoperability, which enables systems to exchange information with unambiguous meaning [169]. However, the study of interoperability standards for CI approaches is a research area that still needs to be fully exploited. The demand for these standards is also growing at a pace never seen before, as the current trend of Industry 4.0 is characterized by complex automation and production systems where CI works with a wide variety of standards, components, and services in a cloud-based environment [170]. Despite such challenges, recent advances have been made in interoperability standards for cloud-based environments. Some examples are the interoperable cloud-based manufacturing system [171], the hybrid manufacturing cloud framework [172], and the new generation artificial intelligence-driven intelligent manufacturing architecture [173].

Another big challenge of CI in integration with ICTs lies in the manufacturing processes of the FSC production stage. Nowadays, CI approaches are usually focused on maximizing accuracy without considering the resources they consume. Nevertheless, with the increasing demand of developing a globally low carbon economy, the need to build an intelligent and environmentally friendly food industry is becoming more urgent [174,175]. In this sense, CI approaches require that a balance be struck between the computational resources they use, the energy they consume, and the performance they can achieve in the time-frame allowed by food manufacturing processes [176]. Furthermore, the aforementioned resources are assets that are not always available in small- and medium-sized food industries. Therefore, more research is required to develop CI methods that are capable of being deployed in IoT devices and smart manufacturing industries under different constraints like energy efficiency and computational resources. In conclusion, this research path could imply bringing in the concepts of edge computing and fog computing [177] for the efficient inclusion of CI in food manufacturing processes [178].

## Figures and Tables

**Table 1:** Summary of CI-based approaches reviewed.

CI Approach	Strengths	Weaknesses
CI-based statistical learning methods	- Expert knowledge of the problem domain where they are applied is not required. - No assumptions about the characteristics of the data available (non-parametric method) are made. - They can work properly with medium and large sized datasets.	- Expert Statistical Learning knowledge is required. - Their performance is highly dependent on the quality and availability of data. - They have problems finding meaningful representations of the data when the complexity of hidden patterns of the data is very high (e.g., computer vision).
Artificial neural networks and Deep learning	- Expert knowledge of the problem is not required domain where they are applied. - No assumptions about the characteristics of the data available (non-parametric method). - They can extract complex and non-linear patterns embedded in data. - Work directly on raw data without almost any need for feature extraction.	- Expert Statistical Learning knowledge is required. - High volumes of data are required. - High computational capabilities are needed.
CI-based optimization methods	- Satisfactory solutions for complex problems. - They can work in scenarios with time and computational capabilities defined by the user.	- They are approximate methods, so an optimal solution is not guaranteed. - Expert knowledge is required for the design of the methods.
Fuzzy systems	- The methods are capable of modeling impressions and vagueness associated with the data of the problem domain. - The results are easily interpretable.	- Expert knowledge associated with the problem domain is required. - Not able to deal effectively with uncertainty associated with the data available.
Probabilistic Reasoning	- Able to deal with high levels of uncertainty in the data available.	- Unable to deal with complex problems characterized by data representing different variables of interest. - Difficulties in modeling ambiguities and inaccuracies in the input data.

## Data Availability

All data analyzed during this study are included in this article.
